# The Unconventional Inspector

**Published:** 1900-08-18

**Authors:** 


### THE UNCONVENTIONAL INSPECTOR.

In the recently issued annual report of the Chief Inspector of Factories and Workshops there is an interesting record of the work done by some of the lady inspectors in laying bare the ramifications of the truck system in the north-west of Ireland. The story of the adventures of these ladies not only shows how long and persistently an abuse like the truck system tends to hang about home industries, which are outside the usual routine of factory work (for it is now close upon seventy years since the Truck Act was passed), but it also puts in a very striking light the advantages which may sometimes accrue by public servants stepping outside the daily routine of official work and introducing a dash of unconventionality into their proceedings. Lady inspectors have again and again shown that it is possible to do something more than merely inspect. They have been able to enter into confidential relations with the women workers and "to invite confidence as from one woman to another," and thus to become cognisant of evils which would never have come under the eye of the ordinary inspector. This may perhaps be called detective rather than inspectorial work, but it is none the less useful. In this way Miss Squire, one of the lady inspectors mentioned, having received reports from various sources of the continued and open violation of the law by the payment of wages in inferior goods in many parts of the north-west of Ireland, determined to note for herself on the spot the extent of the evil, so she went as a private individual, and lived among the people with her eyes and ears open. The reluctance of the Irish peasantry to accept the assistance of an official of the Government, even to free them from oppression, and their dread of being stigmatised as an "informer," make it impossible to obtain from them any information when once the official character of the questioner is known; but a residence of three weeks as a tourist in the centre of the fishing district of The Rosses enabled her, as she says, "to become intimately acquainted with the habits and conditions of work and payment of wages of the peasantry, whose charming courtesy to a stranger taking refuge from wind and rain in their cabins, or joining them along the lonely roads in their long tramps barefoot to the shops for work and groceries, or while herding cattle on the bogs and among the mountains," made conversation about the matters with which she was concerned natural and easy. And what she found was that all the people were smarting under one crushing injustice—that of inability to obtain money payment for their labour. Whether the work were the knitting of socks or the making of shirts, there was one cry everywhere—that they could not get money, but were paid in tea and sugar, which were both dear and bad. Knitting is the great industry. The material is given out by the local shopkeepers, who are agents for the large firms. To obtain this material the women and girls tramp long distances, and then, when the work is done, back they tramp with the finished work to the agent's shop to receive in payment not money, but just what tea, sugar, spirit, or cloth he chooses to give them. All this is absolutely contrary to law, and one may judge what a tyranny it is, and what a hold these agents have over the people, by the difficulty which was experienced in getting evidence as to what was going on. Although the system is well recognised, and is openly spoken of in friendly converse, a new face appears whenever it becomes a question of giving evidence. "Every device is resorted to to escape appearing in court as a witness"; and one need not wonder, for when they did so appear, notwithstanding that it was perfectly well known that they came forward under compulsion, the employers at once refused to give them any more work, and but for the help of a special fund, which had been started for the purpose, some of these witnesses would have been reduced to beggary. Fortunately Miss Squire, "by a carefully prepared stratagem," succeeded on one occasion in being herself a witness to the whole transaction, and saw the socks handed over the counter, the yarn for fresh socks given out, and the packets of tea and sugar given in payment; but the investigation was difficult and trying work, and was attended with hardships and even dangers; and when one thinks of the months during which this lady, living practically under police protection, was engaged in rooting out these evils, witnessing against them, or driving long distances on dark and stormy mornings in November and December in pouring rain to fetch witnesses to the courts, "no car driver in the neighbourhood being available, or willing to incur the odium of aiding 'the Government' by 'bringing in' the witnesses," and when one reads that the system attacked was supported by people who by their shops, their inns, their proprietorship of the cars, represented the wealth and carrying power of the local community, and by their marriage with the priests' and magistrates' families, and in some cases even by their position as magistrates themselves, represented the order of the community, one recognises how very far from mere inspectorial duty has been the work done by Miss Squire, and her colleague, Miss Deane. As the Principal Lady Inspector says, they were during this time "anything but factory inspectors." The work has been well done, and it has been well worth doing; but it has not been done on formal lines, and the account given of the manner in which the attack on this great evil was made by these two ladies is enough of itself to show how advantageous it is that inspectors should sometimes break adrift from office rules, and instead of contenting themselves with the making of "inspections" should set themselves to rummage out the truth, and seek for information wherever or however it may be found.

